# Gas phase multicomponent detection and analysis combining broadband dual-frequency comb absorption spectroscopy and deep learning

**DOI:** 10.1038/s44172-023-00105-z

**Published:** 2023-08-01

**Authors:** Linbo Tian, Jinbao Xia, Alexandre A. Kolomenskii, Hans A. Schuessler, Feng Zhu, Yanfeng Li, Jingliang He, Qian Dong, Sasa Zhang

**Affiliations:** 1 Key Laboratory of Education Ministry for Laser and Infrared System Integration Technology, Shandong University, 72 Binhai Road, Qingdao, 266237 China; 2 Shandong Provincial Key Laboratory of Laser Technology and Application, Shandong University, 72 Binhai Road, Qingdao, 266237 China; 3 State Key Laboratory of Crystal Materials, Institute of Novel Semiconductors, Shandong University, Jinan, 250100 China; 4 Department of Physics and Astronomy, Texas A&M University, College Station, TX 77843-4242 USA; 5 School of Physics and Astronomy, Sun Yat-sen University, Zhuhai, Guangdong 519082 China; 6 Department of Communications and Networking, Xi’an Jiaotong-Liverpool University, 111, Ren’ai Road Dushu Lake Higher Education Town SIP, Suzhou, 215123 China; 7 School of Information Science and Engineering, Shandong University, 72 Binhai Road, Qingdao, 266237 China

**Keywords:** Infrared spectroscopy, Computer science

## Abstract

In absorption spectroscopy, analysis of multicomponent gas mixtures becomes challenging when absorption features overlap (blended spectra). Here we propose a gas sensor which can accurately identify the species and retrieve the concentrations of components in a gaseous mixture in a broad spectrum. The sensor integrates a mid-infrared dual-frequency comb laser source for spectrum acquisition and a deep learning algorithm for spectral analysis. The sensor was tested on gas phase mixtures of methane, acetone and water vapor. A prototype sensor was assessed in realistic scenarios in real time. We also systematically analyzed and presented explicit visualizations to explain the underlying working mechanism of the algorithms.

## Abstract (web summary)

Libo Tian, Jinbao Xia and colleagues introduce a gas sensor that can identify the components and concentrations of gases in a mixture. This method integrates optical sensing with deep learning, providing a solution for complex gas mixture analysis.

## Introduction

The determination of trace gas concentrations and gas species of unknown multicomponent gas mixtures is required for environmental atmospheric monitoring^1^, medical diagnostics^2^ and industrial process monitoring^3^. Optical methods based on infrared spectroscopy are preferred when a fast-response, highly selective and sensitive, non-invasive real-time measurement is required^4,5^ compared with chemical analysis which needs the precise calibration of chromatographic columns, manual sampling procedures, sample preparation and other additional constraints^6,7^. While narrowband lasers are well suited for sensitive detection of a specific molecule, especially when the absorption lines of different molecular species are separated, considering the limited tuning range, the analysis of a gas mixture will likely require several such individual laser sources. and possibly complex optical alignment^8^. Although with the integrated frequency division multiplexing the simultaneous detection of multi-species in gas mixtures^9^ will be possible, still the tuning over the required spectral interval will take considerable time.

A better alternative is to use a broadband laser source covering multiple absorption lines of different gases^1,10–14^. Since molecular absorptions are strongest and highly specific in the mid-infrared (MIR) range, lasers in this region can potentially provide a higher sensitivity. A variety of broadband MIR laser sources have been realized, such as frequency combs (FC)^6,13,15–17^, super-continuum sources (SC)^12^, quantum cascade lasers (QCLs)^1,10^ that were combined with high sensitivity optical methods to further improve the detection limit and robustness of spectroscopic systems. Among the various methods, the dual-frequency comb (DFC) spectrometers integrate the broadband sources and comb teeth resolution into one platform, which exhibits high signal-to-noise ratio in a relatively short acquisition time and shows the potential of multicomponent gas identification in real-time applications such as biomass pyrolysis and combustion diagnostics^18,19^. The DFC system provides a unique set of characteristics that are especially useful for multicomponent gas mixture sensing, combining the broad spectral span, high spectral power, and high spectral resolution with a high spatial coherence, stability and ease of use^20–23^. High impact experiments have demonstrated the advance of FCs for wide variety of molecular species detection for both sharp spectral lines and continues bands^24^. The rapid data acquisition enabled by the DFC spectroscopy reduces the impact of long timescale drifts or fluctuations of experimental considerations on the overall data acquisition^25^ which is highly desirable for time-resolved studies such as chemical kinetics^26^. However, in addition to a high measurement sensitivity, achieving a high specificity often poses an even greater challenge due to overlapping of the absorption spectra of different components^12^. Such an overlapping makes it difficult to directly retrieve the corresponding concentrations from the observed absorption peaks, especially when a large number of gas species are present in the gas mixture sample^27,28^. Moreover, the interference of unknown gases other than the target gases posts another challenge for the detection accuracy.

Modeling of the blended spectra is a feasible way to solve the intrinsic spectral cross-interference problem. Although some decoupling algorithms were proposed, the identification of gas components and computation of their concentrations from the measured overlapping absorption spectra remains a time-consuming challenge. Mathematical methods, such as Bayesian estimation, principal component analysis and linear discriminant analysis were used to determine and analyze the concentrations of various substances^29^. Often, the justification of these methods is based on a series of assumptions as prerequisites, which in reality are not completely met, resulting in low accuracy of the concentration retrieval^29^. The simple least squares (LS) or partial least squares (PLS) methods for fitting of blended spectra to achieve real-time data analysis are particularly favored by researchers^30,31^, but the inherent problem of such methods is that they cannot identify the gas mixture composition associated with the blended spectrum. Although within a selected set of components the best fit still can be found, the question is how extensive this set has to be, since often all the components of the gas mixture to be measured are not known in advance^32^. Consequently, the presence of the spectral contribution of an unidentified gas will inevitably lead to the prediction deviation in the determined concentrations of the selected set of gas components. With the adoption of machine learning and deep learning technology, intelligent algorithms have been widely used in various fields of optical applications, such as gas concentration retrieval^33^, signal filtering^34,35^, ultrashort pulse reconstruction^36^, hyperspectral image and material spectral classification^37^. For the purpose of gas sensing, traditional machine learning algorithms such as K-nearest neighbors, decision tree, random forest and support vector machine have been applied and compared^38,39^, but the results still require considerable improvements. Although deep learning has successfully implemented gas classification^40–43^ and concentration regression^33,35,44^ separately, the cooperation of multiple models complicates and slows down the algorithm computation^29^. Only few studies have recognized the importance of gas species identification and integrated the capabilities of component identification and concentration retrieval into a single model. As such, one model is based on a one-dimensional convolution neural network (1D-CNN)^45^ and another model employs the state-of-the-art double-layer recurrent neural network (RNN) with attention mechanism (2L-ARNN)^46^.

In this work, we propose a multicomponent gas mixture sensor integrating a fast broadband absorption spectrum acquisition and spectral analysis based on a deep neural network algorithm. Further we refer to this system as a multicomponent gas identification and characterization module (MGICM). In terms of the gas species of interest in the present study, we mainly focus on the detection of gas mixtures of the greenhouse gas methane (CH_4_), the typical respiratory marker acetone (CH_3_COCH_3_), and the common interfering gas water vapor (H_2_O). The strong overlapping of the absorption features of these gases obscures or makes hard direct identification of gas components from the spectral information. With respect to the spectral acquisition hardware system, we use a broadband DFC source in conjunction with a home-made multi-pass cell (MPC) realizing high sensitivity and robust optical system (referred to as DFC-MPC) for rapid high precision and resolution broadband absorption spectra acquisition. In addition, we propose an end-to-end spectral analysis model (SAM) based on a multilayer perceptron (MLP), a structure of the neural network to process the blended spectra. By optimizing the generalized loss function, the trainings for the components identification and concentrations retrieval are simultaneously achieved to fulfill the in-situ and real-time requirement. In order to solve the data scarce problem, the architecture tuning and training of SAM are implemented on the dataset established by accurately simulating the absorption spectra of the aforementioned multicomponent gas mixtures with different concentrations and mixing conditions. The well-trained SAM takes advantage of broadband spectra to identify specific spectral patterns rather than focusing on individual narrow spectral features, and thus achieves highly accurate identification of gas components and precise gas concentration retrieval of multicomponent gas mixtures without any preprocessing. To highlight the advantages of the model performance, SAM is evaluated by comparing with the gas mixture recognition models 1D-CNN^45^ and 2L-ARNN^46^. Subsequently, the performance of the complete MGICM integrating DFC-MPC and SAM in a realistic detection scenario is assessed by real-time measurements, which shows much faster speed compared to traditional methods. Moreover, we also compared the measured absorption spectra with simulated ones that use concentrations determined by MGICM and thus evaluated the performance of the whole sensor in in-situ and real-time measurements, the results show that the MGICM can effectively address the challenges of baseline intensity fluctuations, model regression errors and unknown absorbers. Finally, inspired by the application of class activation map^47,48^ for visual classification, we modified and adapted this approach to our context visualizing spectral patterns of blended spectra and discussed their recognition by the considered neural network models.

## Results

### MIR multicomponent gas sensor setup

**Fig. 1:**
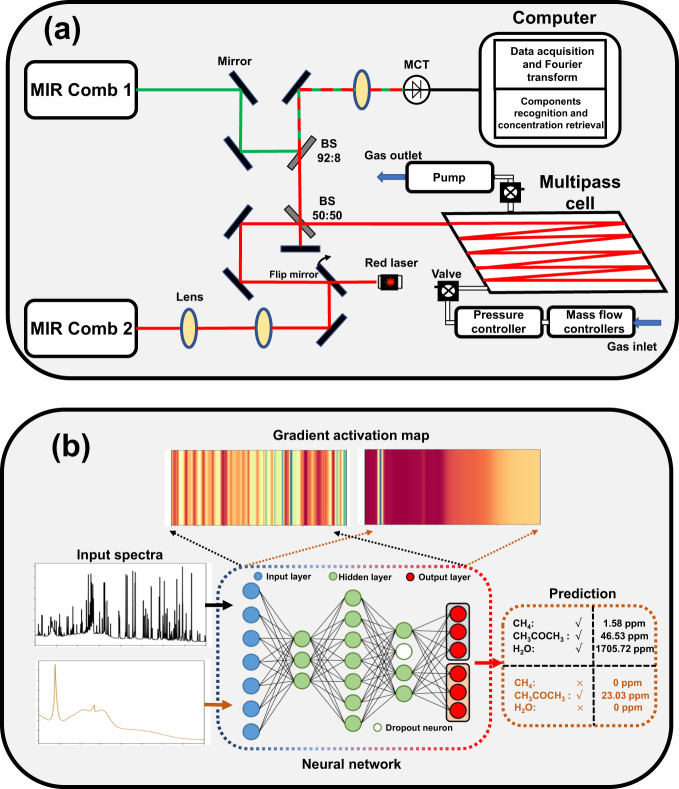
An overview of the proposed multicomponent gas identification and characterization module (MGICM). **a** The layout of the DFC-MPC optical system. The experimental setup includes two MIR comb sources, mirrors and lenses allowing to couple the MIR comb2 into the multi-pass cell (MPC), one 50:50 beam splitter (BS) to split the reference and signal pulses, one 92:8 BS to combine the pulses from the two combs, and an MCT photodetector with computer for data acquisition and blended spectra analysis module. Membrane pumps, a pressure controller and mass flow controllers (MFC) are also employed to maintain stability of measurement conditions. **b** The schematic diagram of the spectral analysis model (SAM). The neural network takes as inputs the measured spectra and outputs the identified components as well as predicted concentrations. The gradient activation maps (GAMs) lend insights of responses of neural network to the spectral patterns.

The MGICM includes a spectral acquisition hardware system DFC-MPC as shown in Fig. 1a, which consists of a DFC laser source, a home-made novel MPC, data acquisition electronic devices, and the analysis model SAM as shown in Fig. 1b, which performs gas component identification and concentrations retrieval.

### Configuration of DFC-MPC

The DFC source has been introduced in detail in our previous work^49^. Two MIR difference-frequency generation (DFG) combs (Menlo Systems, MIR Comb) based on femtosecond Er-doped fiber oscillators are used. The repetition rates are locked at the frequency of ~250 MHz and referred to the Rb frequency standard (Stanford Research, PSR10). The MIR comb1 has ~120 mW output power, covering a spectral range from 2.8 to 3.6 µm (2700 cm^−1^ to 3600 cm^−1^). The pulse duration is ~80 fs. The MIR comb2 employs a higher output power Yb-doped fiber amplifier to generate an MIR comb of ~300 mW with a similar spectrum and pulse duration. We have characterized the DFG MIR combs by measuring their spectra and interferometric autocorrelation traces. The coherence of the MIR combs has been verified by heterodyne beat experiments, as shown in Fig. S1 and Supplementary Note 1. The absorption features in the spectra are due to the water vapor in the laboratory ambient air.

The adopted long-path MPC has also been introduced in our previous work^50^. The volume of MPC is 2.5 L and the radii of curvatures of the two confocal mirrors are 1 m. The optical path with the reflection spot patterns on the mirrors as shown in Fig. S2 are designed to provide the effective path length of 580 m. For the alignment, we first used a visible red diode laser to adjust the multi-pass mirrors. Then the MIR beam replaced the red laser beam by turning up the flip mirror and was aligned to enter the MPC. A more detailed description of the MPC is presented in the Supplementary Note 2.

After mode matching lenses, the MIR comb2 is split by a 50:50 beam splitter. The transmitted (signal) beam is coupled into the MPC while the reflected beam is guided back with a mirror and serves as a reference. The signal pulses exiting from the MPC are recombined with the reference pulses on the same 50:50 beam splitter and overlap with pulses from the local oscillator (LO) MIR comb1 on a 92:8 beam splitter. The combined pulses are aligned and focused on a liquid nitrogen cooled HgCdTe (MCT) detector with a 100 MHz bandwidth (Kolmar Technology, KMPV11-0.1-J1/AC100). By using neutral density filters and beam splitters, the total incident power on the detector is adjusted to be less than 2 mW, above which the detector starts to show signs of saturation, and the powers of signal and reference beams from the MIR comb2 and the power of LO MIR comb1 are roughly equal. The two femtosecond DFG MIR combs were locked with slightly different repetition rates at ${f}_{r1}={{{{\mathrm{249998,633}}}}}$ Hz and ${f}_{r2}={{{{\mathrm{250000,122}}}}}$ Hz, thus the difference is ${{{{{\rm{\delta }}}}}}{f}_{r}=1489$ Hz. An interferogram formed by many pulse pairs with different delays is recorded on the oscilloscope (Tektronix, MDO4104B-3, sampling rate of 250 MPSPS). The maximum record length is 20 Mega points, corresponding to 80 ms, or ~118 complete interferograms. The recorded 80 ms signal is converted from the time domain into the frequency domain by Fourier transform with a simple software phase correction. The high-resolution spectra show more noise in the baseline, especially when normalized with the reference taken at different time. To increase the signal-to-noise ratio (SNR) and save the data acquisition and processing time, the reference and signal interferograms are acquired with interleaving and the spectral resolution is relaxed to match the absorption features.

**Fig. 2:**
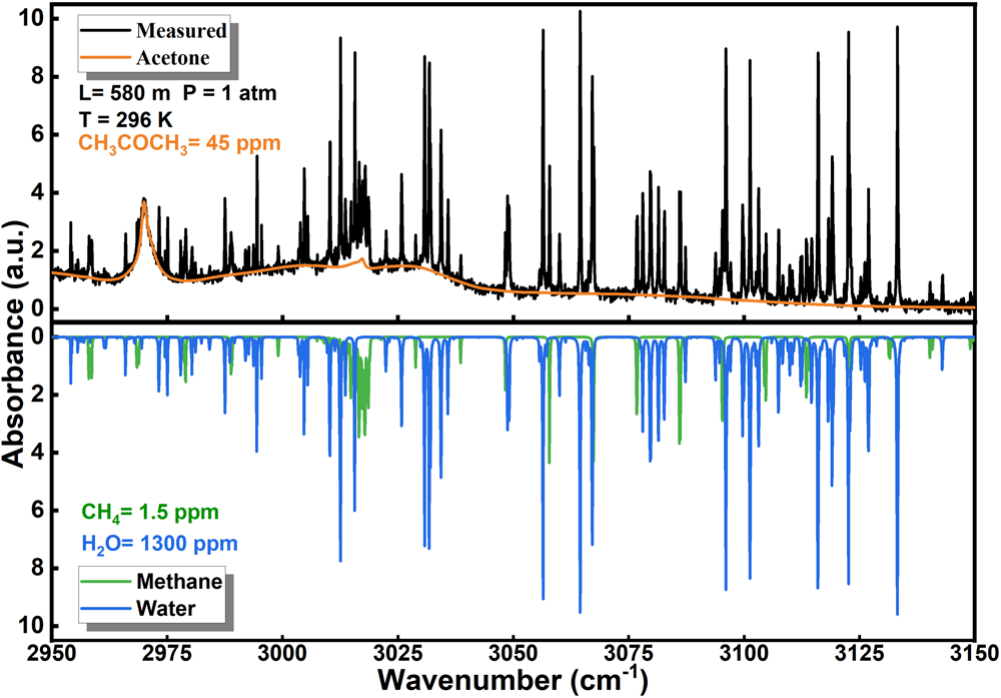
Absorption features of CH_4_, CH_3_COCH_3_ and H_2_O in the selected spectral window. The upper part illustrates the absorbance of CH_3_COCH_3_ (in orange line) and the whole gas mixture (black line) while the lower part displays that of CH_4_ (green line) and H_2_O (blue line). The absorption features of the components overlap and jointly contribute to the absorbance spectrum of the mixture.

Considering spectral location of the molecular vibrational modes and assuring an adequate power spectral density of the DFG combs, the spectral window of 2950 ~ 3150 cm^−1^ is selected as suitable for simultaneous multiple gas species detection. Two rectangular windows of 80 ${{{{{\rm{\mu }}}}}}{{{{{\rm{s}}}}}}$ with apodization centered at signal and reference interferogram peaks are used, resulting in 0.06 cm^−1^ MIR resolution, which is sufficient to resolve the absorption features in ambient air. The good agreement between the normalized spectra of the absorbance and the simulation demonstrates the high precision of the frequency calibration as shown in Fig. 2.

### The principle of laser infrared absorption and the inverse problem

**Fig. 3:**
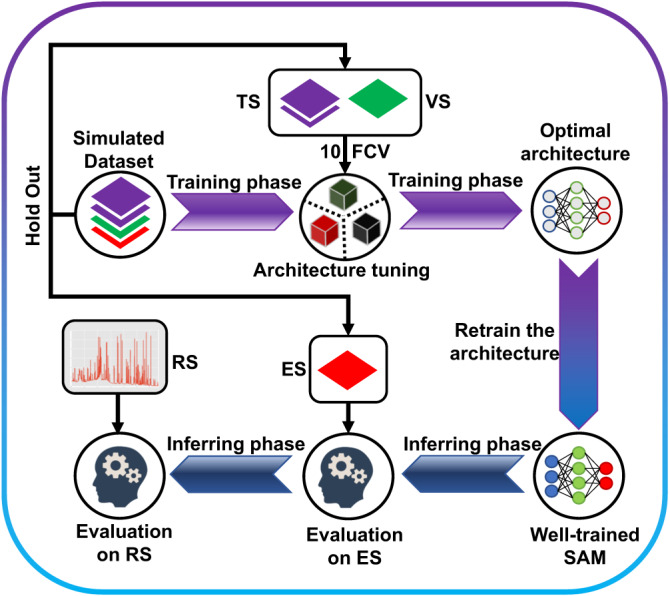
The schematic flowchart of the algorithm. 10FCV: 10-fold cross validation. TS, VS and ES represent the training set, validation set and evaluation set respectively. RS stands for the real-spectra. The ES is initially held out from the dataset, then the model architecture is tuned by TS and VS through 10 FCV during the training phase. The generalization capability of the pre-trained SAM is then verified by the ES and RS in the inferring phase.

The infrared gas sensing techniques are based on the theory of molecular absorption spectroscopy. Following the Beer-Lambert law, for the laser beam at the optical frequency ${{{{{\rm{\nu }}}}}}$ with the initial intensity ${I}_{0}\left({{{{{\rm{\nu }}}}}}\right)$ and the transmitted intensity ${I}_{t}\left({{{{{\rm{\nu }}}}}}\right)$ at the exit from the gas medium the following relation holds

1 $$\alpha \left({{{{{\rm{\nu }}}}}}\right)=-{{{{{\rm{ln}}}}}}\left[\frac{{I}_{t}\left({{{{{\rm{\nu }}}}}}\right)}{{I}_{0}\left({{{{{\rm{\nu }}}}}}\right)}\right]=S(T)\phi ({{{{{\rm{\nu }}}}}})P{c}_{g}L$$

where $\alpha$ is the absorbance, $S(T)$ is the temperature-dependent line intensity of the transition, *P* is the ambient pressure, *L* is the effective optical path length, ${c}_{g}$ is the concentration of a given gas component $g,$ $\phi \left({{{{{\rm{\nu }}}}}}\right)$ is the normalized line-shape function of the molecular absorption. The blended absorption spectrum ${S}_{b}$ of the gas mixture that is formed by $n$ gaseous species can be reasonably represented as the sum of the absorption spectra ${\alpha }_{i}$ of all $i$-th gas components plus the noise term $N$:

2 $${S}_{b}=\mathop{\sum }\limits_{i=1}^{n}\left({\alpha }_{i}|{g}_{i}{{{{{\rm{;}}}}}}{c}_{i}\right)+N$$

where $N$ is the sum of the various noises contained in the sensor system, including thermal noise, shot noise, interference fringe noise and so on; $n$ is the number of gas species to be measured. In the present study *n* = 3 and the index values $i={{{{\mathrm{1,2,3}}}}}$ represent methane, acetone and water vapor, respectively. Figure 2 illustrates the absorption features of CH_4_, CH_3_COCH_3_ and H_2_O in the selected spectral window. The absorption lines of these three gases spread over the whole range and significantly overlap with each other. Therefore, two problems should be solved, first, the unknown components of the gas composition should be determined and, second, the unknown concentrations of these components from the given blended absorption spectrum should be retrieved. Both tasks are simultaneously optimized through our generalized loss function which is introduced in the Methods section. As a data-driven learning algorithm, the neural network needs iterative training on a large number of samples to achieve the correct mapping of the inputs to outputs. The basic concept of neural networks is provided in the Supplementary Note 3. However, obtaining large number of spectral data through experiments is extremely time-consuming and laborious. In order to solve the problem of data scarcity, we first use Eq. (1) and Eq. (2) with parameters provided by HITRAN^51^ and PNNL^52^ databases to accurately compute the blended spectra under various mixing conditions and establish the dataset. The initialized SAM then implements the architecture tuning on the reasonably divided dataset. After determining the optimal model architecture, the well-trained SAM is obtained by retraining on the whole training set (training set + validation set), and its generalization performance is preliminarily evaluated on the evaluation set. Subsequently, the real data obtained from experiments are used to evaluate the performance of the predictive function realized by the SAM. The different steps of the algorithm are shown in the flowchart of Fig. 3.

### Datasets

In the present study, we established the dataset of blended absorption spectra of multicomponent gas mixtures by proper simulation. It has been confirmed that the model which is pre-trained on the simulated dataset can also perform well on the real data, which is especially important in cases where it is difficult and time-consuming to collect experimental data^34,35^. This is due to the good reproduction of the distribution of experimental data by a well-designed simulation. The absorption data of CH_4_ and H_2_O from 2950 cm^−1^~3150 cm^−1^ were obtained from the HITRAN database. According to the experimental conditions (*P* = 1 atm, *T* = 296 K, Path length = 580 m), the absorption spectra of both gases with different concentrations were computed. With spectral resolution of 0.062 cm^−1^ the absorption spectral data consisting of 3321 sampling points were obtained. The CH_4_ concentration was varied randomly in the range of 0~50 ppm, while the concentration of H_2_O was within the range of 1000~2000 ppm, corresponding to the normal indoor humidity of around 4%. Similarly, we downloaded the absorption coefficient of CH_3_COCH_3_ at 1 ppm with a path length of 1 m at room temperature and pressure of 1 atm from the PNNL database, and thereby calculated the absorption spectra of acetone in the concentration range of 0~50 ppm. Besides, the challenges of spectral perturbations to the accuracy of the predictions need to be considered. Compared with preprocessing, such as filtering the measured spectra, the model with capability to handle complex situations is more attractive. Therefore, the output power fluctuation, unknown gas absorption, background noise and non-standard baseline normalization to the absorption dataset were added to force the model to overcome the impact of these factors during training. We constructed a set of spectral data for seven different mixing properties: (1) multicomponent mixture of CH_4_, CH_3_COCH_3_ and H_2_O; dual-component gas mixtures of (2) CH_4_, CH_3_COCH_3_; (3) CH_4_, H_2_O; (4) CH_3_COCH_3_, H_2_O; and single-analyte gas of (5) CH_4_; (6) CH_3_COCH_3_; (7) H_2_O. This set we denoted as ${{{{{\rm{X}}}}}}\in {{\mathbb{R}}}^{m\times {n}_{s}}$, where $m$ = 17500 is the number of spectral samples and ${n}_{s}$ = 3321 corresponds to the vector length of each spectrum. Gas indices, components and quantitative details of seven types of gas mixtures are summarized in Table S1, and the task of gas component identification becomes a multi-label classification problem. The number of gas species $n$ is 3 in our case, therefore we established $2\times n=6$ digit labels referring to the components and concentration information contained in each spectrum, as shown in Table S2. The first three digits are designed as gas component identifiers (CI) to indicate if the specific component is present in the mixture, while the last three digits are used as concentration regressor (CR) to show the concentration of the component. The label is denoted by ${{{{{\rm{Y}}}}}}\in {{\mathbb{R}}}^{m\times 2n}$. Notably, the output layer of SAM is designed to consist of six neurons corresponding to the label. During inference, only when SAM identifies the presence of the specific component, namely the probability of its presence predicted by the CI of SAM is greater than 0.5, then the corresponding concentration will be output by the CR. Otherwise, if the probability of CI is less than 0.5, SAM will set the predicted concentration to zero no matter what the CR predicts. In this way, the problem of disability of determining the cause of extremely low concentration predictions (i.e. whether is due to the low concentration gas or is due to prediction errors without the presence of gas) is eliminated and the reliability and anti-interference ability of SAM is greatly improved compared to the algorithms without gas species recognition capability. We also find that the lower threshold of CI helps to improve the limitation of detection of the system, while limits the ability of the model to identify the spectral contribution of unknown gases. Therefore 0.5 is the tradeoff that takes both factors into account (See Fig. S3). More details about how the CI thresholds effect the model’s performance please see Supplementary Note 4.

### Model architecture optimization

In order to obtain the optimal model architecture that can accurately identify the gas mixture components and determine the corresponding concentrations, 10-fold cross validation (10-FCV) strategy is employed. Firstly, the blended spectra dataset is randomly divided into the training set and the test set according to the ratio of 9:1 following the hold out (HO) method, which ensures the independent and identical distribution (IID) principle of the test set and training set. Subsequently, the training set is again randomly divided into 10 parts, nine of which are taken as the new training set and the remaining one as the validation set. The averaged results of the metrics from performing 10 times the training and validation, are used as the estimation of the model performance under a specific architecture. This process is also illustrated in the training phase in Fig. 3.

With respect to the optimization of training hyper-parameters, we use Bayesian optimization (BO) to carry out an extensive search of batch size, training epochs, learning rate as well as the number of layers. As illustrated in Fig. S3, the hyper-parameters combo with best results (lowest training loss, validation loss as well as best validation accuracy) comes from the combination of batch size of 256, epochs of 500, learning rate of 0.01 and 3 hidden layers. The smaller batch size and longer training period does not significantly improve the performance. The relatively large learning rate has achieved faster and better training effect as was assessed from the loss functions of gas component identification and concentration retrieval.

**Fig. 4:**
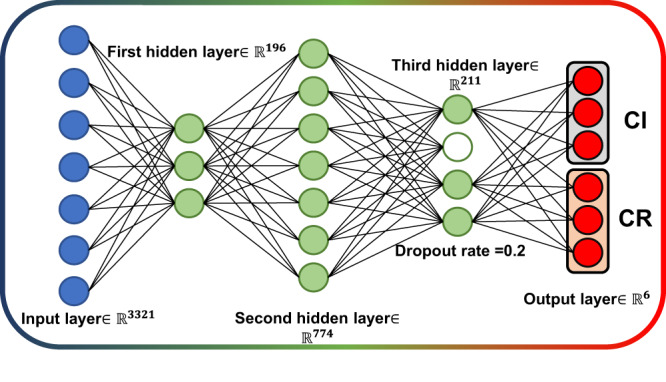
The schematic diagram of the optimal model architecture. The dimension of the input layer corresponds to 3321 sampling points of the input spectrum. The input vector is fed into the neural network from the input layer and then flows through the three hidden layers, and realizes the identification of gas composition and concentration inversion respectively through CI and CR in the final output layer.

The BO is also employed for the optimization of the model architecture, and the number of neurons in each layer has been well swept in the hyper-parameter space. The number of layers varies from 1 to 5 with the number of neurons changing from 2 to 1024. As shown in Figs. S4 and S5, the optimal neural network is a three hidden layer architecture with 196 neurons in the first hidden layer, 74 neurons in the second hidden layer and 211 neurons in the last hidden layer. In addition to the three hidden layers, the neural network also includes an input layer of 3321 neurons and an output layer of 6 neurons. Moreover, we found that the number of neurons in the last hidden layer has greater impact on the performance of the neural network with the validation set than that of the first two hidden layers. Therefore, we have added a dropout layer with dropout rate = 0.2 between the last hidden layer and the output layer to avoid the overfitting. The schematic diagram of the final optimal neural network model is shown in Fig. 4. Once the optimal model architecture and the optimal training setup are determined, the trainable parameters of the optimal model are reinitialized and retrained on the complete training set.

Notably, for a fair comparison, the 2L-ARNN and the 1D-CNN were trained on the same dataset. Traditional non-learning methods have advantages in small, low-noise datasets with strong variable correlation, but struggle with high-dimensional, complex data like gas spectra. Comparisons with traditional algorithms were omitted due to their limitations in simultaneous tasks and poorer performance. Our proposed model, SAM, was solely evaluated against state-of-the-art models. For the 2L-ARNN, an end-to-end encoder and decoder framework is applied to construct the whole architecture. The gated recurrent unit (GRU) which is a typical kind of recurrent neural network (RNN) is used to extract hidden features from gas response signals and enhanced by an attention module to build the encoder part. The decoder takes the context vector compressed by the encoder and tries to analyze the gas components and concentrations. Since source codes are not provided in the original papers^46^, we have reproduced the 2L-ARNN model in strict accordance with the information provided, including all elements, such as encoder, decoder as well as the attention mechanism. In contrast, the 1D-CNN is a one-dimensional deep convolutional neural network based algorithm, which is designed for automatic operation without human interventions and is capable of comprehensively extracting and classifying different features of individual target gases from the raw data^45^. The application context of 1D-CNN is different to that of the present study, so we have properly adjusted its architecture. The broad spectral inputs are different from the multiple independent response signals of the sensor array, so we have removed the concatenation operation behind the first convolution layer and adjusted the convolution kernel size of the second to fourth convolution layers from 3 to 7 to adapt to the vector size of the broad spectra. In addition, we have modified the output layer by adding three new neurons to make 1D-CNN capable of concentration retrieval. All models are implemented with the PyTorch framework. Python 3.7 programming language is used to compile the program on a standard PC with NVIDIA RTX Titian GPU.

## Experimental results and discussion

### Performance evaluation of SAM

The performance of SAM, 2L-ARNN and 1D-CNN in qualitative gas composition recognition and quantitative concentration detection on test set has been assessed and compared. As described in the previous section, the test set contains spectral data which are independent and distributed similarly with the training set. The data on the seven types of gases are present in both sets, so the error assessment caused by class imbalance of data is avoided.

We first conduct assessment of SAM performance for the gas component identification. For the qualitative identification of gas components, exact matching ratio (EMR) and hamming accuracy (HA) are introduced as evaluation metrics to assess the identification capability of the models for different gas species in the spectra. In the case of the EMR, only the predicted CI that is exactly the same as the label CI can be regarded as the correct prediction for each spectral sample. The exact match ratio can be defined as:

3 $${EMR}=\frac{1}{m}\mathop{\sum }\limits_{j=1}^{m}I({g}^{(j)}={\hat{g}}^{(j)})$$

where $I(\cdot )$ is an indicator function, which takes on the value 1 when ${Y}_{{CI}}^{\left(j\right)}$ is exactly equal to ${\hat{Y}}_{{CI}}^{(j)}$ and 0 value otherwise.

However, only considering the EMR is not sufficient for the evaluation of the presence of multi-components in a gas mixture, since EMR regards partially correct gas composition predictions as faults. Therefore, it is advisable to take into account the partially correct results of predictions. The HA measures the proportion of correctly predicted gas components in all multi-label samples and can be defined as:

4 $${HA}=\frac{1}{m}\frac{1}{n}\mathop{\sum }\limits_{j=1}^{m}\mathop{\sum }\limits_{i=1}^{n}I({g}_{i}^{\left(j\right)}={\hat{g}}_{i}^{(j)})$$

Overall, all models performed well in the identification of gas components, as shown in Table 1. The performance of 1D-CNN was relatively inferior with EMR and HA scores of only 94.28 % and 97.32 %, respectively. 2L-ARNN takes the advantage of the attention mechanism to summarize context information, and thereby it performed better than 1D-CNN in gas identification with the EMR of 95.55 % and HA of 97.96 %. However, the EMR (98.56 %) and HA (99.42 %) scores of SAM were the highest, demonstrating that SAM has the stronger capability to identify multicomponent gas species from the blended spectra. Wilcoxon signed-rank tests show that the differences are statistically important (*P* < 0.005), thus the SAM significantly outperformed other models in terms of EMR and HA.

**Table 1:** The exact matching ratio and hamming accuracy results comparison.

	1D-CNN	2L-ARNN	SAM
EMR	94.28%	95.55%	98.56%
HA	97.32%	97.96%	99.42%

The percentage is showing the probability of accurate determinations. The bold values represent the best results.

**Fig. 5:**
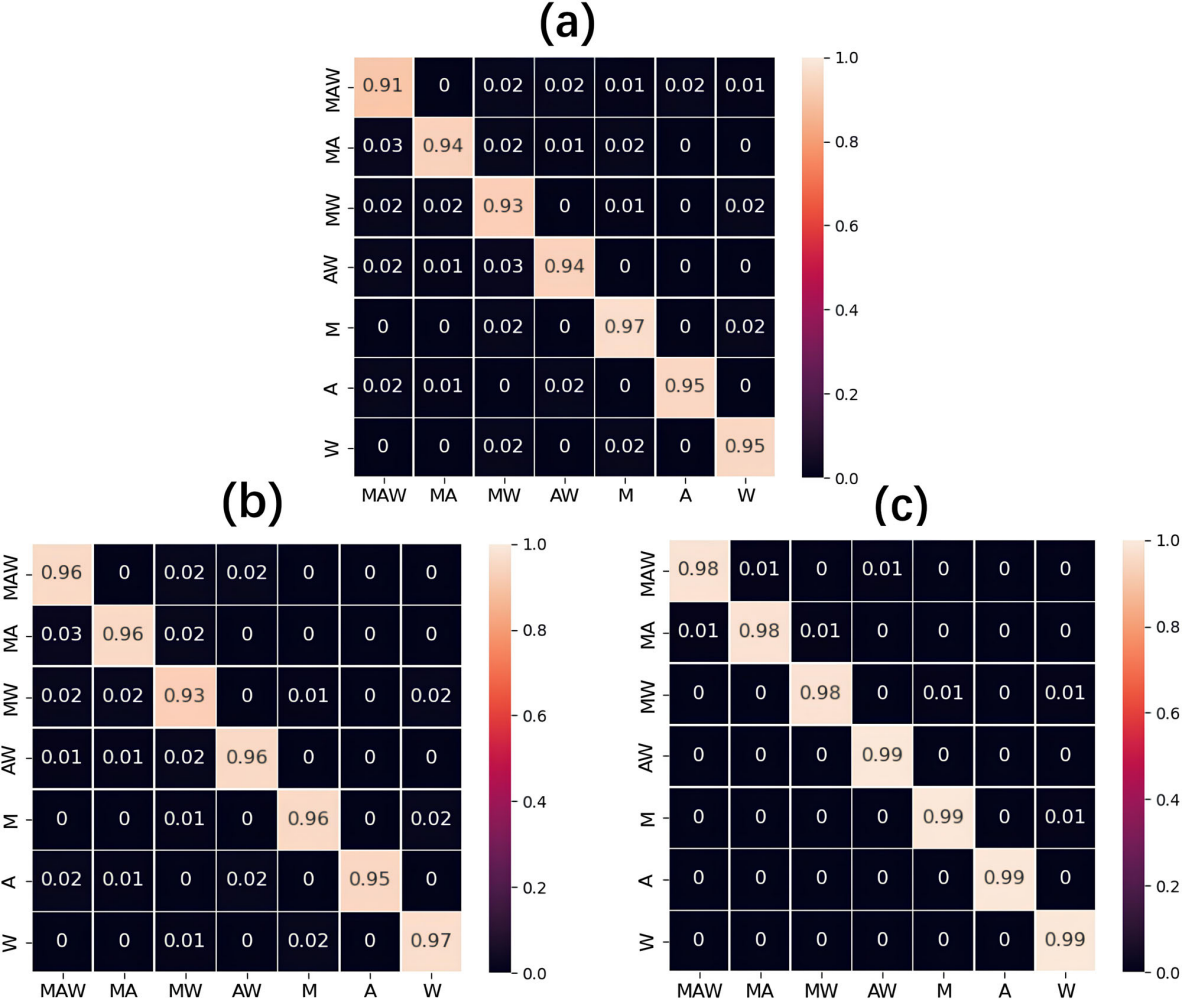
Confusion matrices for detailed gas component identification presentation. **a** 1D-CNN, **b** 2L-ARNN and **c** SAM. The vertical axis represents the true class of the sample while the horizontal axis is the predicted class. Each row represents the probabilities of certain gas compositions determined by the respective model. M, A, and W stand for methane, acetone and water respectively.

Furthermore, we present quantitative measures (confusion matrices) of the gas composition identification from the absorption spectra by different models for seven different types of gas mixtures. The labels along the vertical axis of a confusion matrix (Fig. 5) show the true composition of the sample, while the labels along the horizontal axis correspond to the model-determined compositions. Each row of the matrix represents the probabilities presented with two significant digits of particular gas compositions determined by the models. Thus, the diagonal of the matrix contains the proportions of all the correctly identified compositions. 1D-CNN model has the worst identification rate of only 91 % for the methane, acetone and water vapor mixture (MAW), as shown in Fig. 5a, while the worst performance of 2L-ARNN with only 93 % of correct identification happens for the binary mixture of methane and water vapor (MW), as shown in Fig. 5b. With regard to the single-component gases, 1D-CNN and 2L-ARNN models do not show any advantages, and the identification rate of 2L-ARNN for acetone and that of 1D-CNN for acetone and water vapor was as low as 95 %. The analysis of the identification errors indicates that the prediction failures of models are due to the false identification of the similar absorption features of methane and water vapor. Especially, when the concentrations of methane or water are at extremely low levels, 2L-ARNN and 1D-CNN misjudge the presence of methane or water vapor. In contrast, the overall performance of SAM is relatively stable (see Fig. 5c). The gas identification accuracies of SAM for seven mixture types are maintained at above 98 %, and they are even higher at 99 % for the single-component absorption spectra.

**Fig. 6:**
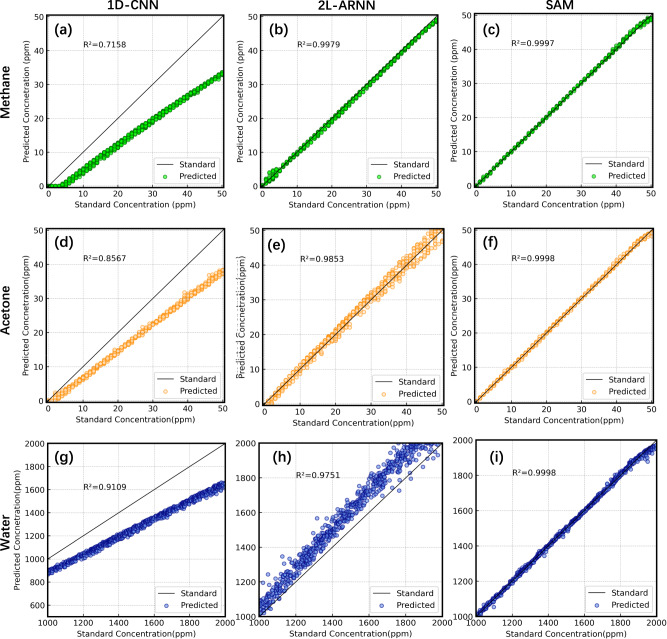
The concentration retrieval results. The concentration retrieval results of different gas species (rows) by different models (columns). **a**–**c** The results represent the concentration retrieval of methane in gas mixture by three models respectively. **d**–**f** The results represent the concentration retrieval of acetone in gas mixture by three models respectively. **g**–**i**, The results represent the concentration retrieval of water in gas mixture by three models respectively

We then conduct the evaluation of the gas concentration retrieval performance of SAM. We have investigated the concentration retrieval capability of the models through the coefficient of determination (${R}^{2}=1-\sum {(c-\hat{c})}^{2}/\sum {(c-\bar{c})}^{2}$) of the gas concentrations ($\hat{c}$) predicted by the models with respect to the standard actual values ($c$) in the test set. As shown in the first column of Fig. 6a, d, g, the concentration retrieval capability of 1D-CNN is the worst, consistent with its component identification results. The concentration predictions of 1D-CNN for three gases are generally lower than the actual gas concentrations even at low concentration levels due to the inability to identify the presence of methane, resulting in methane concentration retrieval value of 0. The ${R}^{2}$ of 1D-CNN is also the lowest among three models, and that of methane is especially low of only 0.72. Besides, the deviation of the predicted results of these three gases from the actual values gradually increases with the rising gas concentrations. Compared with 1D-CNN, the concentration retrieval ability of 2L-ARNN is greatly improved. The prediction capability of methane concentration has even reached a high value of ${R}^{2}=0.9979$, as shown in the second column of Fig. 6b, e, h, while by contrast it still performs poorly for acetone and water vapor. The concentration predictions of water vapor are generally higher than the standard values. The predicted results for the same standard concentrations also show a large variance, indicating the poor stability for the retrieval of acetone and water vapor concentration of 2L-ARNN. Similar to 1D-CNN, such deviation increases with the increase of gas concentrations. In contrast, SAM shows a very high concentration retrieval accuracy for all gases. As shown in the third column of Fig. 6c, f, i, the good agreement proves the highly linear dependence with ${R}^{2} > 0.9979$ for all three gases. Although the results for water vapor show some fluctuations, they are still much better than those of the two other models. Wilcoxon signed-rank tests are also statistically significant with *P* < 0.005 for all cases.

**Fig. 7:**
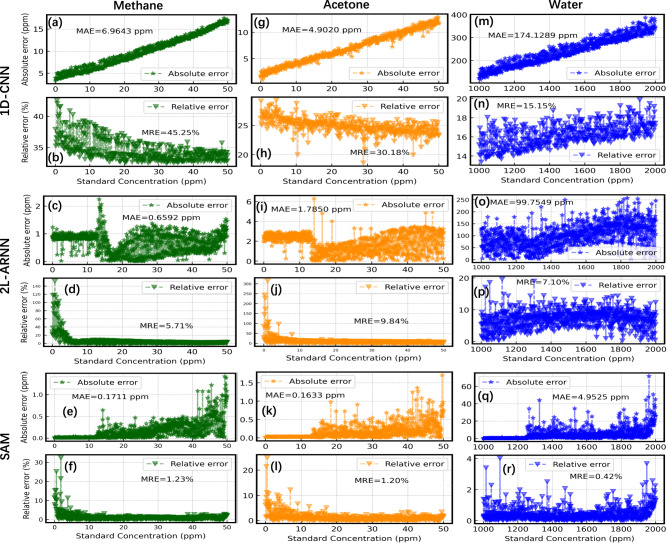
Error analysis of the three models with respect to the three gas species. **a**–**f** The results for methane (green star symbols). **g**–**l** The results for acetone (orange star symbols). **m**–**r** The results for water (blue star symbols). The results for each model are grouped in two rows showing the mean absolute error (MAE) and the mean relative error (MRE).

We have also systematically analyzed the errors of the above concentration retrieval results in terms of the relative error (RE) and the absolute error (AE) as shown in Fig. 7. Consistent with the results of the coefficient of determination, the mean absolute error (MAE) of the predicted results of 1D-CNN for methane and acetone were 6.96 ppm (Fig. 7a) and 4.90 ppm (Fig. 7g), respectively, while the MAE for water vapor is the largest, reaching 174.13 ppm (Fig. 7m). The mean relative errors (MREs) of 1D-CNN for three gases, which are 45.25% (Fig. 7b), 30.18% (Fig. 7h) and 15.15% (Fig. 7n), respectively, are also unacceptably large. For 2L-ARNN, the MAE of methane and acetone are only 0.66 ppm (Figs. 7c) and 1.79 ppm (Fig. 7i), respectively, but the MAE of water vapor reached 99.75 ppm (Fig. 7o), which cannot be ignored. Although the MRE of 2L-ARNN for all three gases is less than 10% over the full concentration range (Fig. 7d, j, p), it is still extremely high for low concentrations of methane and acetone, indicating that the concentration determining capability of 2L-ARNN cannot well adapt to the detection needs of trace amounts of methane and acetone. The MAEs of the predicted concentrations for methane and acetone by SAM are satisfactory, which are as low as 0.17 ppm (Fig. 7e) and 0.16 ppm (Fig. 7k), respectively, and the MREs are kept within 2% (Fig. 7f, l). Since the concentration of water vapor itself is kept at a high level, which is two orders of magnitude higher than that of methane and acetone, the MAE of the concentration prediction of water vapor is relatively large (4.95 ppm, Fig. 7q), while the global RE of SAM for water vapor prediction is not more than 4% and the MRE is only 0.4253% (Fig. 7r). Another notable observation is that although the AE of SAM with respect to all three gas species increases with the increase of the concentrations, the corresponding RE remains almost unchanged, indicating that SAM possesses good detection stability in the full concentration range. Even though the RE of SAM increases in the low concentration range, it is still smaller than that of the other models and remains in an acceptable range. The results of the concentration retrieval for all three models are summarized in Table 2.

**Table 2:** Concentration retrieval results for all models of three gases.

	1D-CNN			2L-ARNN			SAM		
	${R}^{2}$	MAE (ppm)	MRE (%)	${R}^{2}$	MAE (ppm)	MRE (%)	${R}^{2}$	MAE (ppm)	MRE (%)
Methane	0.7158	6.96	45.25	0.9979	0.66	5.71	0.9997	0.17	1.23
Acetone	0.8567	4.90	30.18	0.9853	1.79	9.84	0.9998	0.16	1.20
Water	0.9109	174.13	15.15	0.9751	99.75	7.10	0.9998	4.95	0.43

The bold values represent the best results.

The performance comparison results of the three models are discussed below. The original 1D-CNN does not possess the ability of gas concentration retrieval and our modification may not fully conform to the original intention of its architectural design. Therefore, the results of 1D-CNN with good gas identification performance but poor concentration retrieval performance are reasonable. In addition, a large number of convolution layers in 1D-CNN architecture can indeed provide an abstract feature extraction, but the fitting of complex functions requires more fully connected layers to achieve a satisfactory performance, while the single fully connected layer in 1D-CNN architecture is not suited for this task, which is another cause of its poor performance in concentration determination. The poor performance of the 2L-ARNN model with complex architecture in concentration retrieval is mainly due to its inability to accurately discriminate the similar absorption characteristics of different gas species in the selected optical window range, especially when it is applied to high-resolution broad spectra inputs. Although the GRU reduces the effects of potential gradient dispersion or gradient explosion for the prediction of long sequences, it still does not completely eliminate them. Besides, although the encoder-decoder structure combined with the attention mechanism to some extent overcomes the difficulty of dealing with highly complex feature representation in RNNs, our results show that for the RNN it is still difficult to achieve accurate regression prediction on super-long sequences. By contrast, SAM is satisfactory in both model complexity and performance. The fully connected network architecture enables SAM to adequately approximate the expected predictive function for solving the ill-posed problem according to the universal approximation theorem. The simplified design of SAM also conforms to the compromise between the data fitting and low complexity of the Occam’s razor principle.

### Evaluation of MGICM

**Fig. 8:**
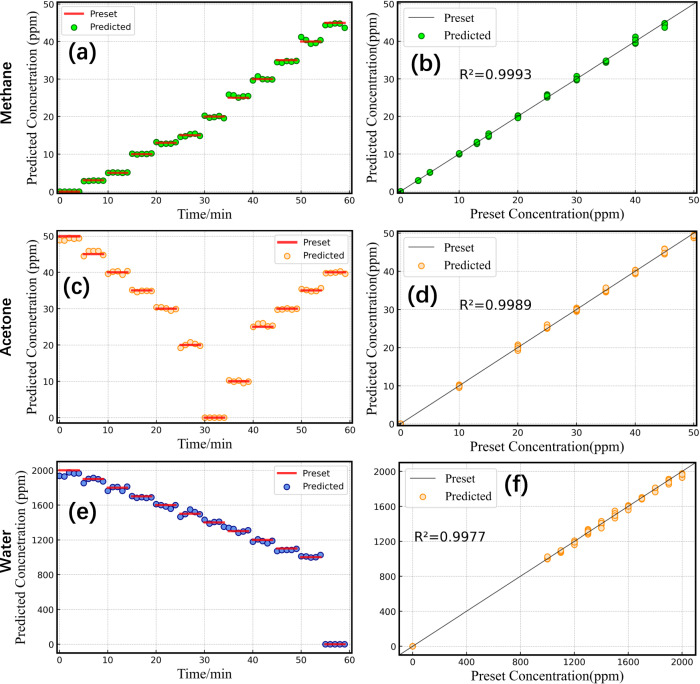
Real-time measurement results of the gas mixture of methane (first row), acetone (second row) and water (third row) for MGICM. The measurement results of step concentration changes for three gases (green circles for methane, orange circles for acetone and blue circles for water. The preset concentrations are shown in read lines) are separately illustrated in (**a**), (**c**) and (**e**). The corresponding coefficients of determination are shown in (**b**), (**d**) and (**f**). The plot of the predicted concentrations was simplified by 1 min interval for clarity. Each interval contains 500 scans, ~0.13 s per scan (80 ms for data acquisition and 50 ms for prediction).

After verifying the performance advantages of SAM in multicomponent gas identification and concentration determination compared with the other models, a feasibility real-time measurement was carried out to demonstrate the capability of the complete MGICM with integrated DFC-MPC and SAM for the measurement of experimental spectra obtained in practical scenarios. In the one-hour experiment, different concentrations of gas mixtures inside the MPC were controlled through the flow rates of MFCs, by stepwise increasing or decreasing the concentration of the three gases with 5 min intervals. The temperature and pressure of the MPC were maintained at 295 K and 1 atm. As illustrated in Fig. 8, the concentrations of individual species of gas mixtures predicted by MGICM are consistent with the preset concentrations (Fig. 8a, b for methane, Fig. 8c, d for acetone, Fig. 8e, f for water). In particular, in the intervals of 0~5 min, 30~35 min and 55~60 min in which the preset concentrations, respectively, of methane, acetone and water were 0 ppm in the gas mixture, MGICM achieved accurate inferences on the composition of gas mixtures. The results show an excellent linearity with ${R}^{2}=0.9993,0.9989,0.9977,$ respectively, for the MGICM over the whole concentration span covering the range expected in a real-time measurement. Small fluctuations in the detection results for the same preset concentrations in the same intervals were observed, which are mainly due to small flow rate changes of MFCs. The good performance of MGICM demonstrates that the pre-trained SAM matches well with the DFC-MPC hardware detection system, and therefore it is feasible for practical applications.

A new experiment was carried out to verify the importance of component identification for gas mixture sensing. two kinds of gas samples were prepared. Taking the methane as an example, we have prepared two kinds of gas mixtures, (a) dual-component gas that only contains water (100 ppm) and acetone (100 ppm), and (b) gas mixture containing the same concentration of water and acetone and a small amount of methane (less than 1ppm). We respectively tested the spectral analyzing results of two models, the original SAM that with the capability of component identification, and its variant that without the capability of component identification, on the two kinds of gas mixtures, and the results were shown in the first two rows of Table 3. For the second gas mixture, there was no difference in the concentration predictions achieved by both models. However, for the first gas mixture, the original SAM can accurately perceive the absence of methane and make a correct concentration output of 0 ppm, while SAM variant incorrectly predicts a small concentration value (0.07 ppm), which is very close to the detection of limit of methane for the proposed system. Such result leads to a problem: If we use a model without the capability of specie identification and get a prediction of the concentration that nears the detection of limit, we cannot determine that this result corresponds to the fact that (1) the gas does not exist, it is just the prediction uncertainty or error of the model, or (2) the gas does exist, and it is indeed of such a small concentration.

**Table 3:** Retrieved concentrations of gas samples by SAM with and without component identification.

	SAM (without component identification)	SAM (with component identification)	PLS
Gas mixture without CH_4_	0.07 ppm CH_4_	0 ppm CH_4_	0.15 ppm
Gas mixture with low CH_4_	0.87 ppm CH_4_	0.87 ppm CH_4_	1.75 ppm
Gas mixture without CH_3_COCH_3_	0.06 ppm CH_3_COCH_3_	0 ppm CH_3_COCH_3_	0.13 ppm
Gas mixture with low CH_3_COCH_3_	0.61 ppm CH_3_COCH_3_	0.61 ppm CH_3_COCH_3_	1.32 ppm

Therefore, we can learn two key points from these cases: (1) regression algorithms do not have the ability of component recognition, (classification models or models with classification capability do), and model prediction error will lead to misjudgment of the causes of low concentration prediction; (2) Model with component identification ability can help distinguish the causes of low concentration results, so as to eliminate the model error at low concentration. Similarly, the experimental results for acetone are summarized in the next two rows in Table 3, and the same conclusion can be conducted. As a representative of the most favored regression algorithm, we added the results of PLS under these two cases, as shown in the third column of the Table 3. PLS was not only unaware of the absence of methane, but the deviation in the predicted values of concentration was also more obvious in both cases.

**Fig. 9:**
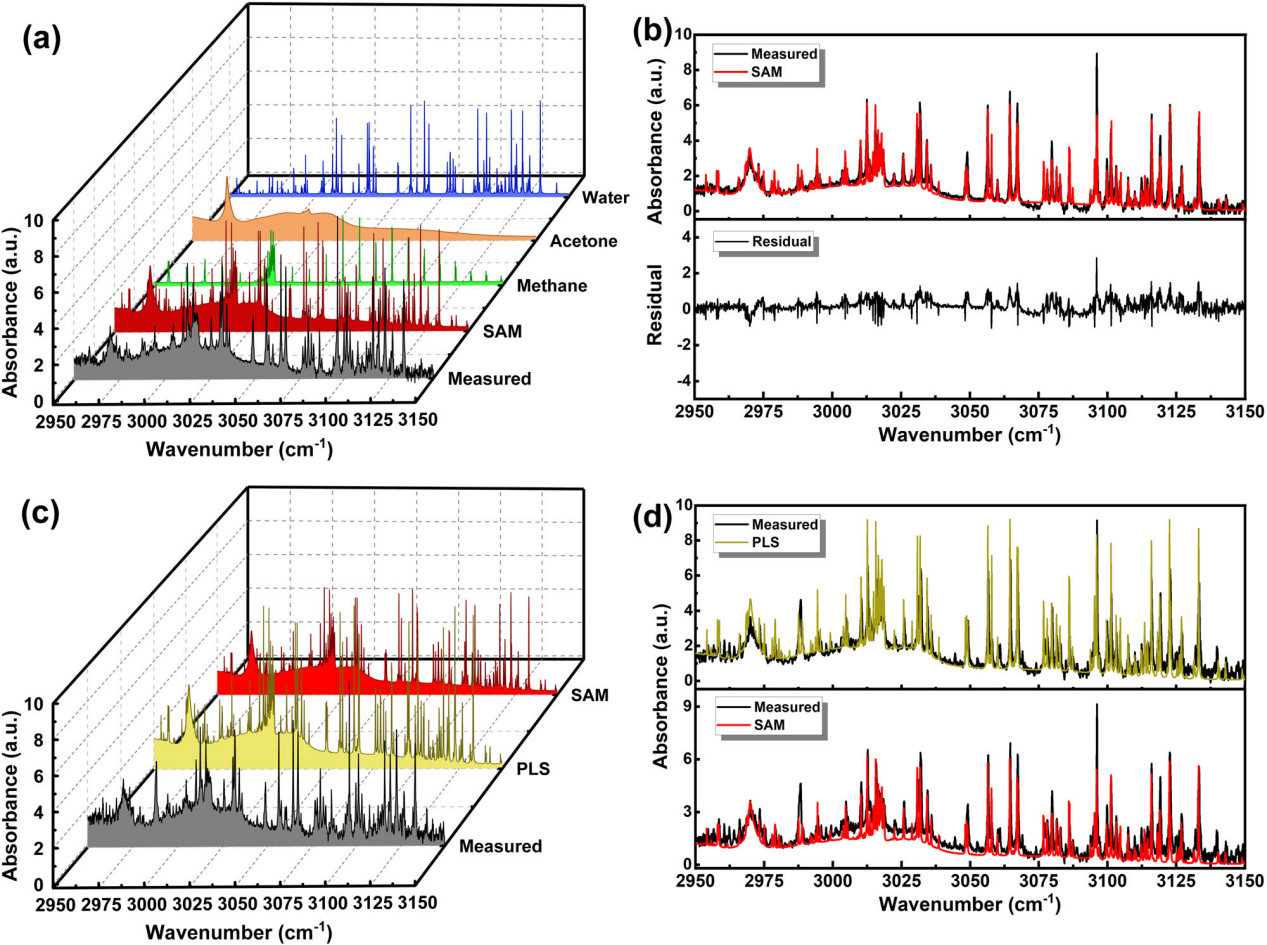
In-situ measurement results of unknown gas mixture. **a** Comparison of the measured spectrum of the unknown gas sample (ambient air and additional acetone) and the spectra of the components computed by the MGICM. The measured spectrum (black line and shade) is obtained directly by the DFC-MPC setup. The SAM spectrum (red line and shade) is synthesized with the retrieved individual components. The measured spectrum is obtained directly by the DFC-MPC setup. **b** Comparison between the measured spectrum and the predicted spectrum (upper plot) and the residual (bottom plot). **c** Comparison of the measured spectrum and predicted spectra of component identification algorithm and non- component identification algorithm of the unknown gas sample (ambient air, additional acetone and ethylene). **d** The comparison between the measured spectrum and the PLS predicted spectrum (upper plot, yellow line and shade) and the measured spectrum and the SAM predicted spectrum (bottom plot). The highlighted regions and discrepancies of the fitted spectra proves the importance of component identification ability. SAM ignores the absorption of ethylene, while PLS increases the concentration of the target gases to fit the additional absorption contribution.

Furthermore, we have carried out the in-situ measurement with MGICM to assess its gas sensing capability in the presence of unknown gases. In addition to the ambient air in the laboratory, we also injected acetone with unknown concentration into the MPC to form a multicomponent gas mixture with unknown species and concentrations. Figure 9a shows the detection results of such a by MGICM. MGICM accurately identified methane, acetone and water vapor in the gas mixture with predicted concentrations of 1.33 ppm, 43.54 ppm and 752.16 ppm, respectively. The sensitivity of the measurement is determined to be 7.6$7.6\times {10}^{-7}{{cm}}^{-1}$ with the SNR about 150, 75 and 120 for water, methane and acetone, and the $3{{{{{\rm{\sigma }}}}}}$ detection limit of the system is estimated to be ~60 ppb. In order to verify the precision of the predictions, we compared the measured spectrum of the ambient air sample with the spectra computed by the MGICM, as shown in Fig. 9b (upper plot). The blended spectrum simulated using the predictions of MGICM showed good agreement with the measured spectrum in general. The absorbance residual in the measured spectral range is basically maintained at a low level, except for the occasional spikes at the positions of narrow absorption lines, see Fig. 9b, bottom plot. Such phenomenon is partly due to the contributions to the absorption spectrum of other unknown trace gases in the ambient air that were not identified by SAM and challenges associated with the air sample, such as additional scattering from airborne particles, the discrete step size of the spectrometer, the limited resolution of the database spectral line and the instrumental noise. Measured mixing ratios can be heavily influenced by the chosen line-shape model as well as changes in the pressure shift and line broadening coefficients for complex gas mixtures. There can be significant deviations when other molecules are added to the gas mixture. These effects could also play a large part in the fit residuals shown in Fig. 9b. Moreover, the residual spikes at the positions of the absorption features of non-target gases also demonstrate that the contribution to the absorbance of non-target components except methane, acetone and water is retained by SAM, which means SAM does not fit the absorption contribution of non-target components in the measured spectrum for the purpose of reducing the residual. Therefore, these residuals imply the existence of other unknown gas species, which illustrates the importance of gas component identification ability by SAM.

Further, we continued to introduce ethylene as interference gas (9 ppm) into the MPC, and compared the result predicted by SAM with that predicted by the non-component identification algorithm PLS, as shown in Fig. 9c, d. Full range absorbance increased due to the contribution of ethylene. Since PLS is not capable to identify gas species, it overestimated the predicted concentrations of the target components to achieve a reasonable fitting (methane 2.19 ppm, acetone 56.76 ppm and water 1163.92 ppm). In contrast, there is an obvious gap between the measured spectrum and the predicted spectra by SAM, indicating that SAM did not wrongly interpret the absorbance of ethylene as an increase in the concentration of the three target gases, especially at 2988 cm^−1^ and 3095 cm^−1^, where the absorption feature peaks of ethylene were not identified by SAM. The results also prove that SAM can effectively solve the challenges of spectral fluctuations. Compared to the normalized absorbance of the N_2_ baseline, the interference of ethylene undoubtedly results in a non-standard normalized spectrum. The influence of the addition of ethylene on the measured spectrum can be clearly seen in Fig. S5 in the Supporting Information. The ability of component identification enables SAM to distinguish the difference between the target gases and the unknown absorbers, ignoring the absorption of ethylene and avoiding the improper deviations of the target species predictions. As shown in Fig. S6. In the upper plot, the absorption spectrum corresponding to the added 10 ppm ethylene is shown and compared with the measured spectrum before adding. The difference in measured spectra before and after adding ethylene can be visually seen in the bottom plot. Ethylene contains continuous multiline absorption over the spectral span, with extensive crosstalk with the three target gases, so the effect on the baseline fluctuation is significant., while SAM still performs well in this case, showing the superiority of algorithms such as PLS that do not have the capability of component identification.

Although SAM is only used at present for the detection of three typical gas components of methane, acetone and water vapor, in view of the success of MGICM and the scalability of SAM, it is completely feasible to apply MGICM for analysis of a more complex unknown gas mixtures by modifying the SAM architecture and expanding the multicomponent gas spectral dataset. Therefore, the proposed combination of SAM and DFC-MPC has high potential in various application scenarios with multicomponent gases, such as environmental monitoring, medical diagnostics and industrial monitoring.

## Spectral patterns and multicomponent gas analysis via deep neural networks

Although we have compared the performance of three well-designed deep learning models for problems of component identification and concentration retrieval of multicomponent gas mixtures, they neither show the transparency of their inner workings, nor they provide the explicit expressions for the predictive functions. Therefore, below we discuss and interpret the mechanism of the implemented deep learning model for our specific task providing also visual illustrations of the obtained results. We use the gradient information flowing into the last hidden fully connected layer of our model to assign the importance values to each neuron for a particular prediction of interest and we call such technique as gradient activation map (GAM). Details of the GAM computation are included in the Methods section.

**Fig. 10:**
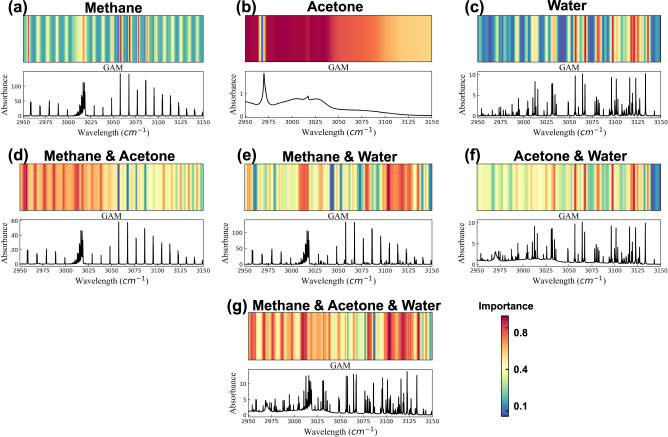
Gradient activation maps (GAMs) of spectral analysis model for seven types of gas samples. single component (**a**–**c**), two components (**d**–**f**), and three components (**g**). The upper parts are the GAMs rendering the spectral responses of neural networks of the various measured gas absorbance spectra as shown in lower parts.

We have found that the change of GAMs of SAM is a process from simplicity to abstraction. The gas component identification and concentration determination of SAM is based on the specific spectral patterns of various mixing proportions that are learned during the training process. Such spectral patterns become more complicated with the increase of the number of gas components as shown in Fig. 10.

SAM shows completely different response for different single-component gases. In terms of methane (see Fig. 10a), SAM pays the highest attention to several characteristic peaks with strong absorption of methane in the position of 2955 cm^−1^and the range of 3050 cm^−1^ to 3090 cm^−1^. Moderate attention is also paid to several obvious characteristic peaks in the range of 3000 cm^−1^ to 3025 cm^−1^, while the responses to other regions with weak or no absorption are very low. Due to the wide spread absorption of water in the spectral window range, the representation of GAM for water is more complex (see Fig. 10c). In addition to the strong responses to absorption peaks that partially overlap with those of methane in the range of 3050 cm^−1^~3075 cm^−1^, there are also distinct responses in the range of 3100 cm^−1^ to 3130 cm^−1^, which are significantly different from the responses for methane in this region. Although water has many characteristic absorption peaks in the range of 2950 cm^−1^~3050 cm^−1^, such weak absorption characteristics are appropriately ignored by SAM. In contrast, the GAM for the acetone absorption spectrum is more predictable (see Fig. 10b), and there are obvious responses to the absorption peaks near 2970 cm^−1^ and 3020 cm^−1^. The GAM response of SAM is similar to the recognition by an experienced spectroscopist, however it is not limited by some specific absorption features, but instead it comprehensively takes the full spectral range into account.

The GAMs of two-component gases are becoming more complex. We consider as an example the GAM of blended absorption spectrum of methane and water mixture (see Fig. 10e), which is different from the case of a single component. In addition to the obvious responses to the independent characteristic peaks of methane and water, SAM pays more attention to the regions from 3000 cm^−1^ to 3025 cm^−1^ and from 3100 cm^−1^ to 3130 cm^−1^ with obvious cross-interferences of methane and water, demonstrating that SAM pays attention to these overlapped absorption regions when inferring the components and concentrations of mixed gases. This is different from the usual method of simultaneous detection of multicomponent gases by human spectroscopist who will avoid the absorption of overlapped areas and will try to find independent absorption features. Similar conclusions can be drawn by observing the GAM of the mixture of methane and acetone and that of acetone and water (see Fig. 10d, f). It also can be found that the dual-component GAMs are not a simple accumulation of single-component GAMs, but a comprehensive consideration of independent characteristic absorption features and blended absorption of the complete spectrum.

When all three gases are mixed, the GAM of SAM is too complex to interpret (see Fig. 10g). More areas that are highlighted in the GAM indicate that more overlapped absorption regions need to be taken into account by SAM. In this case, although we can intuitively see which parts of the spectrum are crucial for the correct inference, it is difficult to trace how the model realizes the extraction of high-level semantics through the spectral information and why. It can be predicted that when the number of gas components to be measured increases, the extraction of spectral semantics by SAM will be even more difficult for explicit interpretation. Nevertheless, the high accuracy of SAM for gas component identification and concentration retrieval still proves that SAM does obtain a set of effective methods through training to solve the specific tasks as presented in this paper without any manual interference. We have applied the same technique to 2L-ARNN and 1D-CNN for visual interpretation, and the GAMs for both models provide us with a clear evidence of their deficiency compared with SAM (as discussed in Supplementary Note 5).

## Conclusions

In conclusion, we have developed the multicomponent gas identification and characterization module (MGICM) which integrates a broadband acquisition method of absorption spectra and spectral analysis algorithm. The spectrum acquisition system referred to as DFC-MPC includes a dual-frequency comb source within the selected spectral window of 2950~3150 cm^−1^ and a home-made multi-pass cell with optical path length of 580 m. A novel multilayer perceptron based neural network model (for short, spectral analysis model or SAM) is proposed and optimized to construct the predictive function for resolving the ill-posed problem of identifying gas species and determining concentrations from the blended spectra. The advantages of the proposed SAM in gas species identification and concentration retrieval were first verified by comparison with the state-of-the-art 2L-ARNN and 1D-CNN neural network models. Consequently, the feasibility of overlapped spectra analysis of the complete MGICM and its robustness to the challenges such as baseline fluctuations, regression error and unknown absorbers in practical detection applications were verified through real-time and in-situ measurements. This proved that the realization of multicomponent gas identification and concentration detection based on broadband light sources combined with the proposed blended spectra processing algorithm that employs deep learning models is an effective and practical approach.

In this work the performance of MGICM was demonstrated on only three specific gases (methane, acetone and water vapor) with their mixtures of unknown compositions showing significant overlap of absorption spectra in the investigated region 2950~3150 cm^−1^. The current SAM is only a prototype, which is used as a benchmark for simultaneous identification and content determination of multicomponent gases. The extension of the proposed approach to gas mixtures with a significantly larger number of components still, as a prerequisite, will require a preliminary screening and selection of a limited set of possible constituent components. The results of this work show the way how the identifying of the unknown components that constitute blended spectra and determining their concentrations can be accomplished simultaneously, and we note that to achieve positive results in such an analysis in previous studies the gas compositions had to be specified in advance. Such multicomponent gas analysis can find applications in environmental monitoring, surveillance of industrial processes and medical diagnostics that involve the need for sensing and analysis of multicomponent gas mixtures. Besides, benefits from a set of characteristics, the DFC can quickly capture the whole spectrum as it changes due to chemical reaction, which is not possible with tunable systems. We note that the combination of DFC and deep learning analysis algorithm is a promising approach for monitoring and quantification of fast chemical reaction kinetics, especially when a variety of reaction products are involved.

The integration of uncertainties from hardware resources into the model is challenging, as these uncertainties are experiment-specific and difficult to experimentally obtain under different configurations. Although our model performs well in specific setups, its generalizability to other devices may be limited. Besides the impact of uncertainties in experimenters’ operations and instrument performance on the model’s training. Errors in the Mass Flow Controller can lead to biased results. We have acknowledged these issues and hope to carry on such work in the future.

## Methods

### Multi-objective optimization with generalized loss function

We achieve simultaneous optimization of both tasks with one loss function by setting the objective to minimize the generalized loss function presented by a combination of the term for the gas component identification combined with the term for the concentration retrieval:

5 $${{{{{{\mathcal{L}}}}}}}_{{obj}}=\mathop{{{{{{\rm{arg }}}}}}{{{{{\rm{min }}}}}}}\limits_{W}({{{{{{\mathcal{L}}}}}}}_{{iden}}+{{{{{{\rm{\lambda }}}}}}\,\cdot\, {{{{{\mathcal{L}}}}}}}_{{con}})$$

where the gas component identification loss term ${{{{{{\mathcal{L}}}}}}}_{{iden}}$ is constructed by binary cross entropy loss, as a metric of model for gas component identification prediction. The gradients obtained from this term guide the trainable parameters $W$ to be optimized in the direction of more accurate identification of gas components. ${{{{{{\mathcal{L}}}}}}}_{{iden}}$ is expressed as follows:

6 $${{{{{{\mathcal{L}}}}}}}_{{iden}} 	=-\frac{1}{m}\frac{1}{n}\mathop{\sum }\limits_{j=1}^{m}\mathop{\sum }\limits_{i=1}^{n} \left\{{g}_{i}^{\left(j\right)} \cdot {{{{{{\rm{log }}}}}}}_{e}\left({\hat{g}}_{i}^{(j)}\right)+\left(1-{g}_{i}^{\left(j\right)}\right)\cdot {{{{{{\rm{log }}}}}}}_{e}\left(1-{\hat{g}}_{i}^{(j)}\right)\right\} \\ 	=-\frac{1}{m}\frac{1}{n}\mathop{\sum }\limits_{j=1}^{m}\mathop{\sum }\limits_{i=1}^{n}\left\{{g}_{i}^{\left(j\right)}\cdot {{{{{{\rm{log }}}}}}}_{e}\left({{NN}}_{{CI}}({X}^{\left(j\right)}{{{{{\rm{;}}}}}}W)\right)+\left(1-{g}_{i}^{\left(j\right)}\right)\cdot {{{{{{\rm{log }}}}}}}_{e}\left(1-{{NN}}_{{CI}}({X}^{\left(j\right)}{{{{{\rm{;}}}}}}W)\right)\right\}$$

where ${g}_{i}^{\left(j\right)}$ and ${\hat{g}}_{i}^{(j)}$ represent the true and predicted presence probability of $i$-th gas composition for the $j$-th spectrum. ${{NN}}_{{CI}}(\cdot )$ is the neural network prediction with respect to the CI based on the trainable parameter matrix $W$ and the given input spectrum ${X}^{\left(j\right)}$. We have explained the significance of the trainable parameters to the neural network and the basic concept of the typical neural network MLP in the Supplementary materials. The term of the gas concentration retrieval ${{{{{{\mathcal{L}}}}}}}_{{con}}$ is the simple mean square error loss function:

7 $${{{{{{\mathcal{L}}}}}}}_{{con}}	 =-\frac{1}{m}\frac{1}{n}\mathop{\sum }\limits_{j=1}^{m}\mathop{\sum }\limits_{i=1}^{n}{\left({c}_{i}^{\left(j\right)}-{\hat{c}}_{i}^{(j)}\right)}^{2}\\ 	=-\frac{1}{m}\frac{1}{n}\mathop{\sum }\limits_{j=1}^{m}{\left({c}_{i}^{\left(j\right)}-{{NN}}_{{CR}}({X}^{\left(j\right)}{{{{{\rm{;}}}}}}W)\right)}^{2}$$

where ${c}_{i}^{\left(j\right)}$ and ${{NN}}_{{CR}}(\cdot )$ respectively represent the actual and predicted CR with the gas index $i$ of the $j$-th spectrum. In addition, we have experimentally found that compared with the gas component identification, the gas concentration retrieval is much harder. Therefore, we set the scaling factor ${{{{{\rm{\lambda }}}}}}$ for the item of concentration retrieval loss such that it forces the model to find a tradeoff between both tasks during training and ensures that the loss function does not converge prematurely before the model can effectively extract the concentration information from the spectra. We empirically tried various values and found that when ${{{{{\rm{\lambda }}}}}}=100$, the training process keeps a good balance between the optimization of both tasks. The learning algorithm used is the Adam optimizer with constants ${\beta }_{1}=0.5$ and ${\beta }_{2}=0.999$.

### Gradient activation map

For SAM, the input tensor gradually becomes more abstract from the input layer to the output layer, and the output of the deeper layer is the advanced semantic extraction of the information of the input tensor itself. From this perspective, we can expect the last hidden fully connected layer to have the best comprehensive representation of the high-level semantics of the components and concentrations. Therefore, we use the gradient information flowing into the last hidden fully connected layer of our model to assign the importance values to each neuron for a particular prediction of interest. Recalling ${a}^{\left(L\right)}\in {{\mathbb{R}}}^{211}$ is the activation of the last hidden layer $L$, the prediction score $\hat{y}\in {{\mathbb{R}}}^{6}$ thus can be computed as:

8 $$\hat{y}=\mathop{\sum}\limits_{n}{{W}_{n}^{x}}^{(L)}\mathop{\sum}\limits_{x}{a}^{\left(L\right)}=\mathop{\sum}\limits_{n}\mathop{\sum}\limits_{x}{{{W}_{n}^{x}}^{(L)}\cdot a}^{\left(L\right)}$$

where ${{W}_{n}^{x}}^{(L)}$ is the weight connecting the location x in the last hidden layer with the prediction of the $n$-th neuron in the output layer. In this case the gradient of $\hat{y}$ with respect to the activation ${a}^{\left(L\right)}$ is the weight assigned to the last hidden layer and the output layer, so the weights directly indicate the importance of the activation, which leads to the prediction of an input spectrum. Intuitively, based on prior work^35^, the output from the last hidden layer should be activated by specific spectral patterns which contain relevant information. Thus, the activation map ${{{{{\rm{M}}}}}}=\mathop{\sum}\limits_{n}{{{W}_{n}^{x}}^{(L)}\cdot a}^{\left(L\right)}$ is the map of the presence of this visual pattern. In addition, the guided backpropagation gradients ${g}_{b}=\frac{\partial \hat{y}}{\partial X}$ provide the fine details about the input spectra $X$. By fusing the guided backpropagation gradients with the activation map a differential guided gradient activation map (GAM) ${g}_{a}={g}_{b}* {{{{{\rm{M}}}}}}$ is created. As a result, important regions of the spectrum which correspond to any inference of interest are presented with spectrum-scaled resolution reflecting the crosstalk of multicomponent gases.

### Supplementary information

Supplementary informatin

## Data Availability

The data that support the findings of this study are available from the authors on reasonable request.

## Code availability

The code that supports the findings of this study is available in the https://github.com/Popsama/Neural-network-multi-component-gas-mixture-analysis-with-broadband-dual-frequency comb.
