# Supplementary material for: Gas phase multicomponent detection and analysis combining broadband dual-frequency comb absorption spectroscopy and deep learning
Source: Commun Eng. 2023 Aug 1;2:54. doi: 10.1038/s44172-023-00105-z (PMC10955922; doi:10.1038/s44172-023-00105-z)
Supplement: Supplementary file 1 — Supplementary informatin [file 44172_2023_105_MOESM1_ESM.pdf]

Supporting information for

Neural network multi-component gas mixture analysis with broadband dual-frequency comb absorption spectroscopy

Linbo Tian[1, 2, †], Jinbao Xia[3,†,*], Alexandre A. Kolomenskii[4], Hans A. Schuessler[4], Feng Zhu[5], Yanfeng Li[3], Jingliang He[3], Qian Dong[6, *], Sasa Zhang[7, 1, 2, *]

[1] Key Laboratory of Education Ministry for Laser and Infrared System Integration Technology, Shandong University, 72 Binhai Road, Qingdao 266237, China

[2] Shandong Provincial Key Laboratory of Laser Technology and Application, Shandong University, 72 Binhai Road, Qingdao 266237, China

[3] State Key Laboratory of Crystal Materials, Institute of Novel Semiconductors, Shandong University, Jinan,250100, China

[4] Department of Physics and Astronomy, Texas A&M University, College Station, Texas 77843-4242, USA

[5] School of Physics and Astronomy, Sun Yat-sen University, Zhuhai, Guangdong 519082, China

[6] Department of Communications and Networking, Xi'an Jiaotong-Liverpool University, 111, Ren'ai Road Dushu Lake Higher Education Town SIP, Suzhou 215123, China

[7] School of Information Science and Engineering, Shandong University, 72 Binhai Road, Qingdao 266237, China

Supplementary Note 1. Characterization results of the DFG frequency combs:

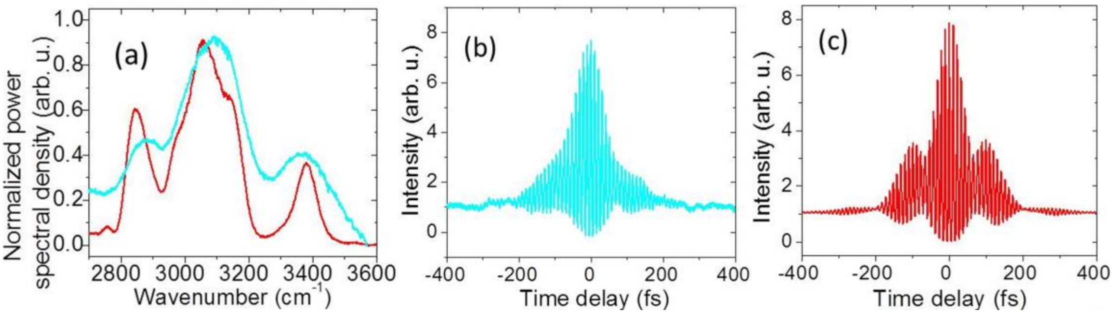

Figure. S1. (a) The spectra measured with a scanning monochromator for MIR comb1 (cyan) and MIR comb2 (red). (b) Interferometric autocorrelation traces for MIR comb1 (cyan) and (c) for MIR comb2 (red).

The DFG sources have a passive carrier-envelope offset (CEO) frequency stabilization. Indeed, since the pump and signal fields originate from the same source, the generated idler field is carrier-envelope phase slip free. The MIR frequency combs are stabilized by stabilizing the source repetition rates.

Supplementary Note 2. Detailed introduction of the multi-pass cell:

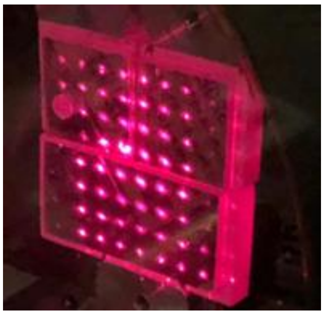

Figure. S2. The aligned spot distributions on the mirrors of the multipass cell.

The multipass cell has a confocal design, the radii of curvatures of the two 50×50 mm^2 mirrors are 1 m and equal to the distance between these two mirrors. Both mirrors are divided into 3 parts, two 25×25 mm^2 squares, and one 25×50 mm^2 rectangle. In one of the square parts, a hole of 5 mm diameter serves as the entrance and exit for the laser beam. The subsequent spots are aligned using the corresponding mirror mounts according to the design. After all six mirror parts are aligned, the spot patterns are formed on the mirrors (Fig. S2), such that the laser beam exits the multipass cell after bouncing between the mirrors with 579 reflections, thus the effective path length is ~580 m. The mirror coating has two high reflection regions, one is in the MIR range (>99.85%, 3100~3600 nm) and the other is in the red visible range (>99.9%, 670~680nm) (Layertec GmbH). For the alignment purpose, we use a visible red diode laser (as shown in Fig. 1(a)) to adjust the multipass mirrors and to achieve the simulated spot patterns. Then the MIR beam replaces the red laser beam by moving up the flip mirror and is aligned through two pinholes to enter the multipass cell. The output of the multipass cell is around 1.0~2.0 mW, depending on the humidity because of the water vapor absorption.

Supplementary Table 1. Simulated absorption spectra datasets:

Table S1 Compositions of different classes of gases and numbers of spectra used in training, validation and evaluation sets.

	Components	Total number of simulated spectra	Number of spectra used for training & validation	Number of spectra in evaluation set
Class 1	CH_4,CH_3COCH_3,H_2O	2500	2253	247
Class 2	CH_4,CH_3COCH	2500	2254	246
Class 3	CH_4, H_2O	2500	2237	263
Class 4	CH_3COCH_3,H_2O	2500	2254	246
Class 5	CH_4	2500	2254	246
Class 6	CH_3COCH_3	2500	2257	243
Class 7	H_2O	2500	2260	240

Supplementary Table 2. 6-digits multi-labels:

Table S2. 6-digit labels. The first three digits are gas component identifier, while the last three digits are concentration regressor. The check marks for particular components show that some concentrations of them are present in the mixture and then the corresponding CI is 1, while if they are not present the CI is 0 and the corresponding position in the regressor has (-).

Class	Component Identifier			Concentration Regressor		
	Methane	Acetone	Water	Methane	Acetone	Water
Class1	1	1	1	√	√	√
Class2	1	1	0	√	√	-
Class3	1	0	1	√	-	√
Class4	0	1	1	-	√	√
Class5	1	0	0	√	-	-
Class6	0	1	0	-	√	-
Class7	0	0	1	-	-	√

Supplementary Note 3. Multi-layer perceptron (MLP) concept

MLP was used to model the predictive function for finding gas composition and concentrations. MLP is a type of neural network with strong nonlinear mapping ability using feedforward network topology which is composed of stacked layers. The neurons inside each layer are closely connected to form the main structure of the neural network. To go from one layer to the next, a set of neurons in each layer computes a weighted sum of their inputs from the previous layer and passes the results through a non-linear activation function as expressed by the following equations:

$$z^{(l)} = W^{(l)} \cdot a^{(l-1)} + b^{(l)} \quad (1)$$

$$a^{(l)} = g(z^{(l)}) \quad (2)$$

where $z^{(l)}$ represents the training parameter matrix $W^{(l)}$and bias vectors $b^{(l)}$ of the hidden l-th layer and the hidden vectors $a^{(l-1)}$ of the linear output of the activation function from the previous layer. $g(\cdot)$ represents the nonlinear activation function. We use the Rectified Linear Unit (ReLU) to replace the sigmoid function to introduce the nonlinear mapping capability for the neural network and thus $a^{(l)} = \max(0, z^{(l)})$. The information is passed layer by layer in MLP and the whole

network can be seen as a composite function that maps the input vector to the final output.

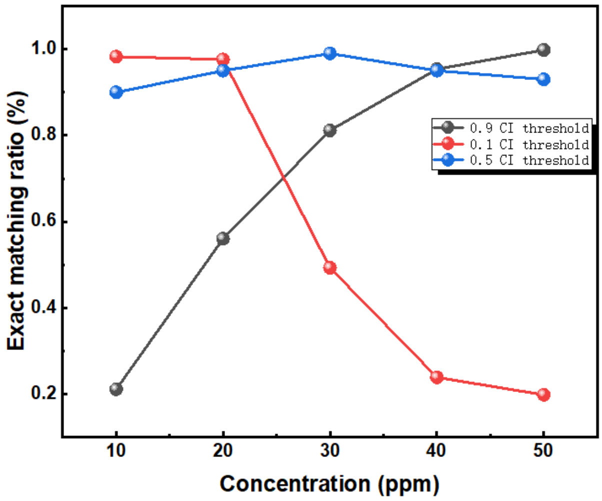

Figure S3. The effect of the CI threshold values on the accuracy of component identification (methane).

Supplementary Note 4. Effect of CI threshold on model performance:

The threshold of the CI does not have a direct relationship with the detection limit, but rather affects the judgment of the presence or absence of gases under different concentration conditions, thereby affecting the detection limit. Specifically, if the threshold of the CI is high, the model will only make a determination of the presence of a gas if there is a significant absorption feature of the specific gas in the measured spectrum (i.e., at a high concentration) that the model can identify with high confidence, and only when the model recognizes the gas as present, will the concentration of the gas predicted by the model be outputted. This makes the model insensitive to low concentrations when the CI threshold is high. Conversely, if the CI threshold is low, the model will decide of the presence of a gas even if the concentration of the gas is very low, as long as the model thinks that there is a possibility of the gas being present. This results in the model being very sensitive to small changes in the spectrum caused by low concentrations, but it can also lead to poor noise robustness.

The Fig. S3 shows the accuracy of the model with different CI thresholds in determining the presence of methane under different concentration conditions. The CI threshold of 0.5 performs well throughout the concentration range.

Supplementary Figure S4. Training hyperparameters optimization:

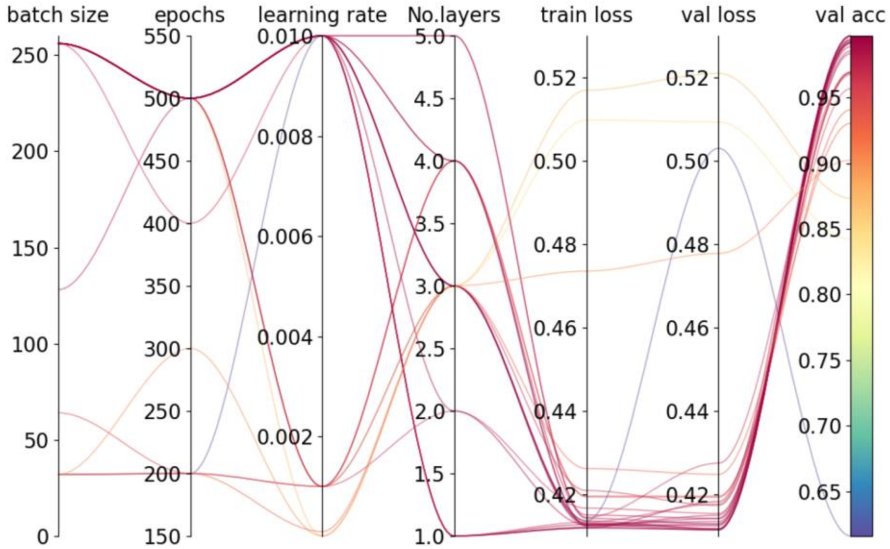

Figure. S4. **Training hyper-parameter tuning results with respect to the batch size, epochs, learning Rate and number of hidden layers.** Different pathways corresponding to different sets of the optimization parameters result in different validation accuracies (the scale on the right). The best results (the lowest training and validation losses as well as the best validation accuracy) come from the combination of the batch size of 256, the number of epochs of 500, the learning rate of 0.01 and 3 hidden layers.

<u>Supplementary Figure S5. Model architecture optimization:</u>

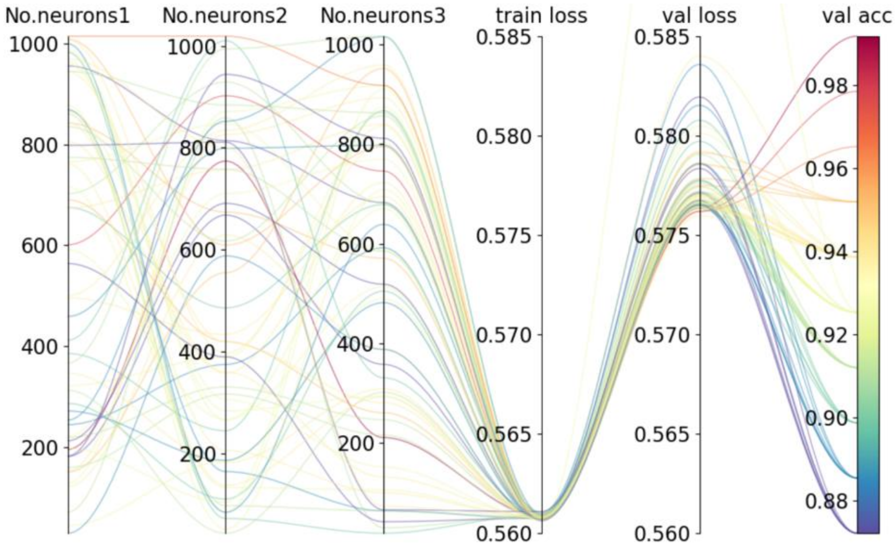

Figure. S5. **Architecture sweeping results with respect to the number of neurons in each hidden layer.** Shown different pathways with different sets of parameters result in different validation accuracies (the scale on the right). The best results (the lowest training and validation losses as well as the best validation accuracy) come from the combination of 196 neurons in hidden layer 1, 774 neurons in hidden layer 2 and 211 neurons in hidden layer3.

<u>Supplementary Figure S6. The effect of addition of ethylene on baseline:</u>

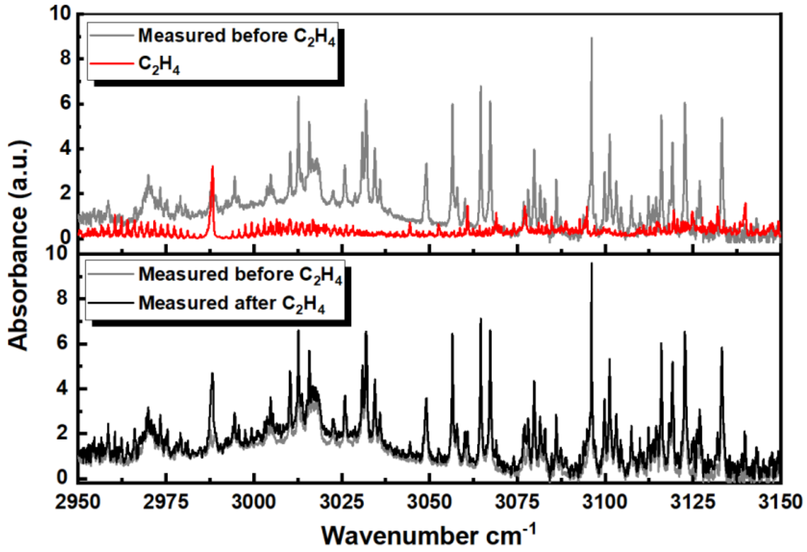

Fig. S6. The effect of addition of ethylene on the original baseline. Comparison between the measured spectrum before C_2H_4 introduced and the C_2H_4 absorbance spectrum of 9 ppm (upper plot) and comparison of spectra before and after adding ethylene (Bottom plot).

Supplementary Note 5. GAMs for 2L-ARNN and 1D-CNN:

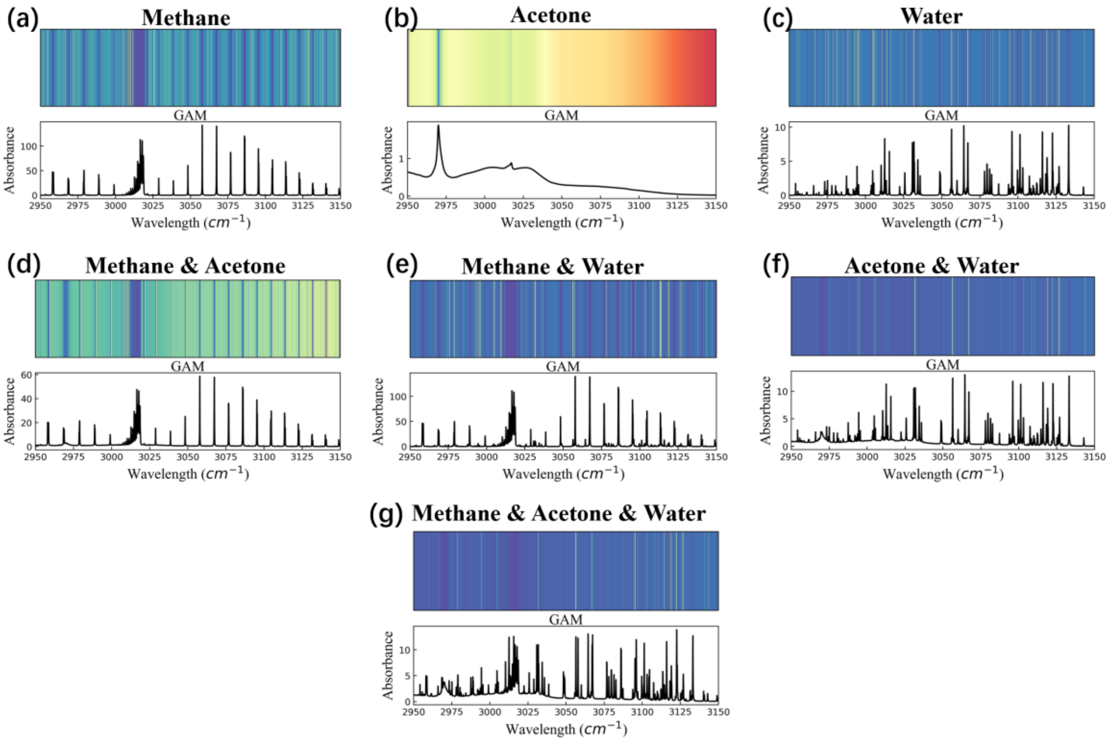

Figure S7. 2L-ARNN GAMs of single component of (a)~(c), and of dual-components of (d)~(f), and of multi-components of (g).

The GAMs drawn by the attention layer of 2L-ARNN to the attention weight of the input spectrum allocation help us understand how 2L-ARNN treats the absorption spectrum and makes the final prediction. As shown in the Fig. S7, the response of 2L-ARNN's attention mechanism to the fine-grained information flow between the input spectra and the prediction is similar to that of our model. The GAM of single component shows that 2L-ARNN can also well capture the

absorption feature peaks of different gases. When the overlapped absorption features occur due to the increase of gas concentration, 2L-ARNN also realizes the detection of multicomponent gases by putting more attention weights into the blended absorption area. Therefore, 2L-ARNN has a good ability to identify multicomponent gases. The difference is that the attention weights are normalized by the attention layer through the softmax function before outputting, so the sum of the attention weight assigned to each position of the spectrum is 1. Such operation leads to the slight fluctuation of each position of the spectrum, which will greatly affect the overall understanding of the model for the spectrum. Therefore, we can see that when detecting the methane and water mixture, although the effective component identification can be carried out through the learned spectral features, the accuracy of 2L-ARNN for water concentration inversion decreases sharply due to the cross interference of methane absorption and water. Moreover, 2L-ARNN takes the dot product of the attention weights and the temporal hidden states outputted by the GRU layer as the context vector to realize the fusion of information and passes it to the subsequent network structure. However, the spectral spatial information extracted by the attention mechanism (i.e., the different responses of the model to different unknown spectra) is consequently discarded, which is also the reason for the poor concentration inversion accuracy of 2L-ARNN compared with our model.

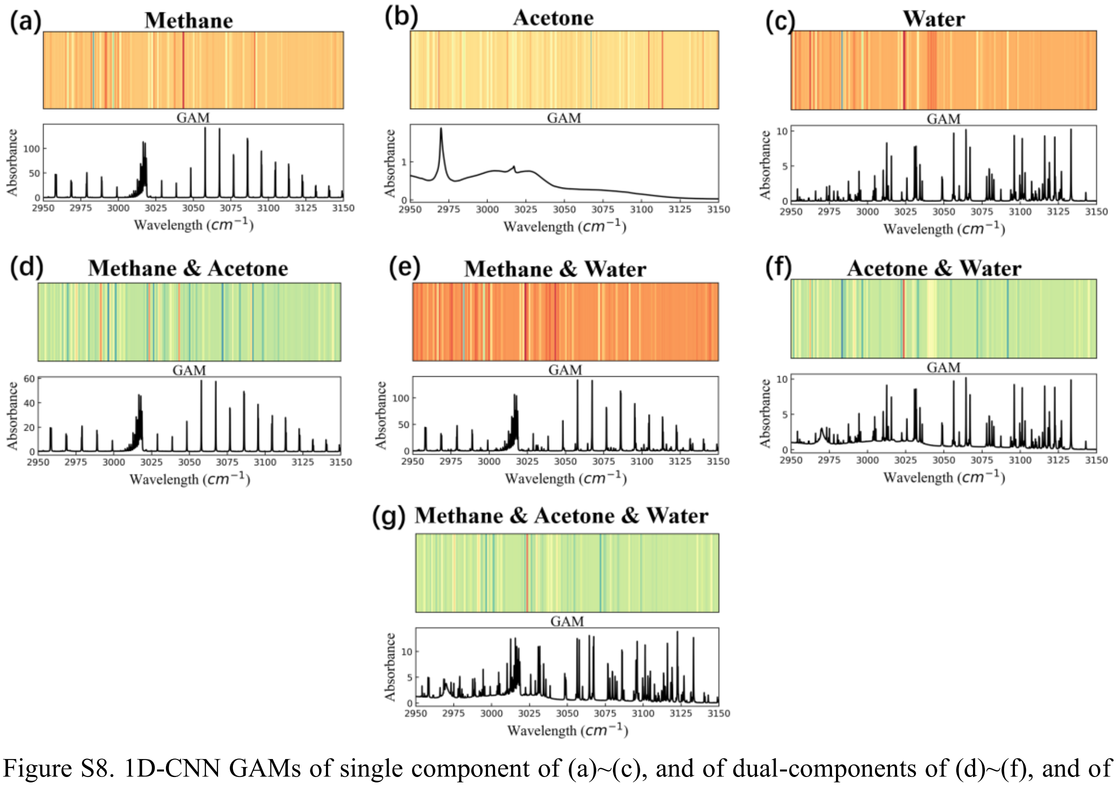

Figure S8. 1D-CNN GAMs of single component of (a)~(c), and of dual-components of (d)~(f), and of multi-components of (g).

For 1D-CNN, as shown in the Fig. S8, the GAMS of the 1D-CNN model do not follow the expected rules. Unlike our model and 2L-ARNN, it seems to pay more attention to the non-absorption region, but does not show obvious response to the absorption characteristic peak. We assume that, on the one hand, due to the performance of 1D-CNN architecture itself, it is difficult for 1D-CNN to learn spectral patterns similar to our model and 2L-ARNN from complex spectral features. Thus this model needs to concentrate more on gas component identification and component concentration inversion, instead of focusing on non-absorption positions to determine gas

components. This drawback leads also to large deviations in the gas concentrations inversion Generally, absorbance provides the bulk of information for calculating gas concentrations, and the first two models also focus on characteristic absorption peaks or aliasing absorption to realize gas concentration inversion. On the other hand, the original 1D-CNN model was not proposed for the gas concentration inversion task, and our modification may have not quite optimal design for this purpose.
